# pH-Responsive Liposomes of Dioleoyl Phosphatidylethanolamine and Cholesteryl Hemisuccinate for the Enhanced Anticancer Efficacy of Cisplatin

**DOI:** 10.3390/pharmaceutics14010129

**Published:** 2022-01-05

**Authors:** Hassan Shah, Asadullah Madni, Muhammad Muzamil Khan, Fiaz-ud-Din Ahmad, Nasrullah Jan, Safiullah Khan, Muhammad Abdur Rahim, Shahzeb Khan, Meser M. Ali, Mohsin Kazi

**Affiliations:** 1 Department of Pharmaceutics, Faculty of Pharmacy, The Islamia University of Bahawalpur, Bahawalpur 63100, Pakistan; hasanshah342@gmail.com (H.S.); muzamilpharmacist@gmail.com (M.M.K.); nasrullahjan14@gmail.com (N.J.); safiullahkhan856@gmail.com (S.K.); muhammadabdurrahim88@gmail.com (M.A.R.); 2 Department of Pharmacology, Faculty of Pharmacy, The Islamia University of Bahawalpur, Bahawalpur 63100, Pakistan; fazi2670@gmail.com; 3 Department of Pharmacy, University of Malakand, Chakdara 18800, Pakistan; shahzebkhan@uom.edu.pk; 4 Department of Pharmaceutical Sciences, School of Health Sciences, University of KawaZulu Natal, Durban 4041, South Africa; 5 Department of Neurosurgery, Henry Ford Hospital, 2799 West Grand Boulevard, Detroit, MI 48202, USA; mali8@hfhs.org; 6 Department of Pharmaceutics, College of Pharmacy, King Saud University, P.O. Box 2457, Riyadh 11451, Saudi Arabia; mkazi@ksu.edu.sa

**Keywords:** cisplatin, pH-responsive liposomes, MDA-MB-231 cell lines, SK-OV-3 cell lines, confocal scanning laser microscopy

## Abstract

The current study aimed to develop pH-responsive cisplatin-loaded liposomes (CDDP@PLs) via the thin film hydration method. Formulations with varied ratios of dioleoyl phosphatidylethanolamine (DOPE) to cholesteryl hemisuccinate (CHEMS) were investigated to obtain the optimal particle size, zeta potential, entrapment efficiency, in vitro release profile, and stability. The particle size of the CDDP@PLs was in the range of 153.2 ± 3.08–206.4 ± 2.26 nm, zeta potential was −17.8 ± 1.26 to −24.6 ± 1.72, and PDI displayed an acceptable size distribution. Transmission electron microscopy revealed a spherical shape with ~200 nm size. Fourier transform infrared spectroscopic analysis showed the physicochemical stability of CDDP@PLs, and differential scanning calorimetry analysis showed the loss of the crystalline nature of cisplatin in liposomes. In vitro release study of CDDP@PLs at pH 7.4 depicted the lower release rate of cisplatin (less than 40%), and at a pH of 6.5, an almost 65% release rate was achieved compared to the release rate at pH 5.5 (more than 80%) showing the tumor-specific drug release. The cytotoxicity study showed the improved cytotoxicity of CDDP@PLs compared to cisplatin solution in MDA-MB-231 and SK-OV-3 cell lines, and fluorescence microscopy also showed enhanced cellular internalization. The acute toxicity study showed the safety and biocompatibility of the developed carrier system for the potential delivery of chemotherapeutic agents. These studies suggest that CDDP@PLs could be utilized as an efficient delivery system for the enhancement of therapeutic efficacy and to minimize the side effects of chemotherapy by releasing cisplatin at the tumor site.

## 1. Introduction

Cancer remains one of the leading causes of mortality, and more than eight million people die due to cancer each year [1]. The World Health Organization estimates that the number of new cancer cases might increase from 11.3 million in 2007 to approximately 15.5 million in 2030. Chemotherapy is one of the treatment options for cancer in addition to surgery and radiotherapy. The delivery of chemotherapeutic agents has low concentration at the tumor site, inevitable distribution, and widespread side effects, which limits its clinical applications. Therefore, the targeted delivery of chemotherapeutic agents has become a focus of scientific research to deliver the drug at the site of action [2,3].

In recent years, nanotechnology has become a scientific buzzword for drug delivery research. Nanoparticulate drug delivery systems are attributed to nano-sized (10–200 nm) drug delivery systems that may facilitate the targeted delivery of a drug at the tumor site. The targeting of these therapeutics agent(s) has bought a massive revolution in cancer chemotherapy via the effective delivery of cytotoxic agents at the tumor site [4].

Vesicular drug delivery systems are highly ordered assemblies consisting of one or more concentric bilayers formed by the self-assembly of amphiphiles upon hydration [5]. Among the vesicular drug delivery system, liposomes have been extensively investigated as a carrier of choice for the delivery of both hydrophilic and lipophilic therapeutic agent(s). Liposomes are bilayered vesicles of phospholipids enclosing a hydrophilic core [6]. Liposomes have shown promising results in the delivery of chemotherapeutics to the site of action as they resemble cell-membrane structure and display biocompatibility, low immunogenicity, enhancement of half-life, safety and efficacy. However, conventional liposomes can only achieve delivery to the initially targeted organs/tissues, and there is still a certain inevitable distribution and damage to the normal organs/tissues [7]. To overcome the aforementioned limitations of conventional liposomes, stimuli-responsive liposomes have been fabricated.

Stimuli-responsive drug delivery systems are those agent(s) that undergo a physical/chemical change in response to a stimulus. The field of stimuli-responsive drug delivery systems has investigated the concept of a pH-responsive drug delivery platform(s) [1,8]. The effectiveness of pH-responsive drug delivery systems is based on the fact that they can exploit well-characterized pH differences between blood and pathological conditions (such as infection, inflammation and cancer), and also between certain intracellular compartments such as cytosol, endosome, and lysosomes in our body [9].

Cisplatin (CDDP), chemically known as cis-diamminedicholoroplatinum (II) anticancer agent, is used in the treatment of various malignancies such as breast, ovarian, testicular, cervical, bladder, head and neck, brain and non-small- cell lung cancers [10,11]. It acts as DNA cross-linking agent and interferes with the replication and transcription of DNA synthesis. It is the most widely used anticancer agent due to its broader efficacy in the treatment of various types of tumors [12]. The broader applications of CDDP are limited by resistance, rapid inactivation, and severe side effects (nephrotoxicity, neurotoxicity, and myelotoxicity) [13]. Therefore, to overcome these CDDP-related issues, efforts have been made to develop a CDDP delivery system by using pH-responsive liposomes.

pH-responsive drug delivery platforms offer the potential of enhancing the therapeutic efficacy and minimizing the side effects of chemotherapy by releasing the encapsulated drug at the site of action [14]. The lower pH is a hallmark of tumor/malignancy caused due to excessive metabolite (lactic acid, carbon dioxide, increased expression, and activity of vacuolar-type (V-type) H+-ATPases (proton pumps) [15].

The delivery of a chemotherapeutic agent to the tumor site by the pH-responsive liposomes presents an efficient means of overcoming the problem of targeted drug delivery to the tumor site. Fan, Y. et al., 2017, [2] and Leite, E.A. et al., 2012, [16] reported similar studies that showed improvements of the pH-responsive targetability of liposomes at the tumor site. In the present study, pH-responsive lipid(s) were used that protonate at lowered pH, resulting in the rapid destabilization of the carrier and the release of the drug in the acidic tumor microenvironment [17,18].

## 2. Materials and Methods

### 2.1. Materials

Cisplatin was received as a kind gift sample from Pharmedic Laboratories Pvt (Ltd.) Pakistan. Dioleoyl phosphatidylethanolamine (DOPE) was obtained from Avanti Polar Lipids, Inc., Alabaster, AL, USA, cholesteryl hemisuccinate (CHEMS) from Avanti Polar Lipids, Inc., Alabaster, AL, USA, and DSPE-PEG_2000_ was a kind gift from LIPOID, Steinhausen, Switzerland. 4, 6-diamidino-2-phenylindole dihydrochloride (DAPI) was purchased from Vector Laboratories, Inc, Burlingame, CA, USA. Fluorescein isothiocyanate (FITC) and paraformaldehyde were procured from Sigma-Aldrich, St. Louis, MO, USA. Dulbecco modified eagle medium (DMEM) was purchased from Thermo Fisher Scientific Corporation, San Francisco, CA, USA. Polycarbonate-membrane-based mini extruders (Nano-sizer Mini^®^) were purchased from T&T scientific, Knoxville, TN, USA. Chloroform and methanol were of analytical grade and were purchased from Sigma-Aldrich, Steinheim am Albuch, Germany. Distilled water was freshly prepared by the distillation plant (IRMECO^®^, Schwarzenbek, Lütjensee, Germany).

### 2.2. Preparation of Cisplatin Loaded pH-Responsive Liposomes

Cisplatin-loaded pH-responsive liposomes (CDDP@PLs) were prepared via the thin-film hydration method. Briefly, DOPE, CHEMS and DSPE-PEG_2000_ were weighed (Table 1) and dissolved in a 15 mL solvent mixture of chloroform and methanol (2:1, *v/v*) in a round bottom flask. The organic solvent was removed using a rotary evaporator (Heidolph, Schwabach, Germany) under reduced pressure at 75 rpm and 60 ± 2 °C for 3 h. The flask was removed and kept overnight in an oven to remove the solvent residues completely. Then, the lipid film was hydrated using cisplatin solution in phosphate-buffered saline (pH 7.4) (5.0 mg/10 mL). The resultant liposomal suspension was vortexed and sonicated (ELMA, E-30 H, Pforzheim, Germany) for about 10 min (at 25 °C and an amplitude of 30%), and then extruded through polycarbonate-membrane-based mini extruders (100 nm, Nano-sizer Mini^®^, T&T scientific corporations, Knoxville, TN, USA) to obtain the liposomes for further analysis [19].

### 2.3. Physicochemical Characterization of Liposomes

#### 2.3.1. Particle Size, Polydispersity Index (PDI) and Zeta Potential

The particle size, PDI and zeta potential of the developed CDDP@PLs were analyzed using Zeta Sizer-ZS90 (Malvern, Worcestershire, UK). The dynamic light scattering technique was used for the determination of particle size, PDI and zeta potential. The measurement was performed at 25 °C and in triplicate for each sample [20].

#### 2.3.2. Entrapment Efficiency

The entrapment efficiency (E.E) of the CDDP@PLs was determined by the indirect method. Briefly, the liposomes were centrifuged by ultra-centrifugation (Sigma-Aldrich, Darmstadt, Germany) at 12,000 rpm for 40 min. The supernatant was collected for the quantification of the unentrapped drug and the process was repeated in triplicate for each sample. The drug was then estimated by taking absorbance through a UV/Visible spectrophotometer (IRMECO, 2020, Schwarzenbek, Lütjensee, Germany) [21]. The E.E was determined by the following formula:

(%) E.E = (Total amount of drug used-Amount of unentrapped drug)/(Total amount of drug used) × 100(1)

#### 2.3.3. Transmission Electron Microscopy (TEM)

The surface morphology of the CDDP@PLs was determined via transmission electron microscopy (JEOL, 2100, Jeol, Akishima, Tokyo, Japan). The sample was applied on the coated side of the grid and was allowed to settle for 5 min. The grids were then blotted on filter paper and stained with a 1% aqueous solution of phosphotungstic acid and kept for 3 min. The grids were then rinsed with distilled water to wash off the excess stain and then dried at room temperature. The grids were then placed in a sample inlet chamber of TEM and observed, and suitable images were taken at different magnifications [22].

#### 2.3.4. Fourier Transform Infrared (FTIR) Spectroscopic Analysis

Fourier transform infrared spectroscopic analysis is an efficient and accurate technique to find any interaction among the formulation components. FTIR spectra of cisplatin, DOPE, CHEMS, DSPE-PEG_2000_, and CDDP@PLs were measured by using ATR-FTIR (Bruker, Tensor 27 Series, Berlin, Germany) in the range of 400 cm^−1^ to 4000 cm^−1^ [23].

#### 2.3.5. Differential Scanning Calorimetric (DSC) Analysis

DSC analysis was performed to evaluate any possible interaction and to check the physical state of cisplatin in the developed formulation. The differential scanning calorimetric (DSC) analysis of cisplatin, CHEMS, DOPE, DSPE-PEG_2000_ and CDDP@PLs was analyzed via a differential scanning calorimeter (DSC-250, TA instruments, New Castle, DE, USA). In the analysis, the calibration was carried out by using indium for the source of temperature and heat flow. Samples were placed on one pan, and another aluminum pan was used as a reference. The samples were then heated over the temperature range of 25–400 °C [24].

#### 2.3.6. In Vitro Release and Kinetic Modeling

The in vitro release study of CDDP@PLs was performed in USP type-II dissolution apparatus (paddle) using the dialysis bag method. The dialysis membrane of MWCO 12–14 kDa was used. The drug release study was performed for all five formulations in phosphate-buffered saline (PBS) (pH 7.4, 6.5 and 5.5) in dialysis bags at 37 ± 0.5 °C and 70 rpm. All the formulations contained 5 mg of cisplatin. At selected time intervals, 3 mL of release media was collected and replenished with an equal volume of fresh media. The amount of cisplatin released was determined using a UV/Visible spectrophotometer at 210 nm [25]. The data were applied to the kinetic modeling using Zero order, First order, Higuchi, and Korsmeyer–Peppas models [26]. The values of the regression coefficient (R^2^) and release exponent (n) were analyzed for the mechanism of drug release from CDDP@PLs.

### 2.4. Cell Lines and Cell Culture

#### 2.4.1. Cell Lines

Human breast adenocarcinoma cancer cell lines (MDA-MB-231) and human ovarian cancer cell lines (SK-OV-3) obtained from American Type Culture Collection (ATCC) (Manassas, VA, USA) were cultured in a flask containing Dulbecco’s modified eagle medium (DMEM) supplemented with 10% fetal bovine serum (FBS) and antibiotics (100 IU/mL streptomycin). The cells were stored in the incubator with a supply of 5% CO_2_ and at 37 °C, and passage was performed after 80% confluence.

#### 2.4.2. Cytotoxicity Study

The cytotoxicity studies were performed on MDA-MB-231 and SK-OV-3 cells via an MTS (5-(3-carboxymethoxyphenyl)-2-(4,5-dimethyl-thiazolyl)-3-(4-sulfophenyl) tetrazolium assay (colorimetric technique). An MTS assay is based on the conversion of tetrazolium salt into a colored formazan by the mitochondria activity of living cells. The amount of formazan depends on the viable cell count in the culture and is measured with a spectrophotometer. Cells previously cultured were seeded in each well of 96-well plates. After 24 h of incubation, cells were treated with cisplatin solution and liposomal formulation at a cisplatin concentration range of 0.078 to 10 µg/mL. The absorbance of the solution was measured on a microplate reader (BioTek, Winooski, VT, USA) at 490 nm [27].

#### 2.4.3. Cell Uptake Study

##### Fluorescence Microscopy

The qualitative cellular uptake study was performed using a confocal scanning laser microscope. Cells (5000) were seeded on coverslips in a 6-well plate containing 2 µL of media for two hours. Cells were placed in the incubator with the supply of CO2 and allowed to adhere for 24 h. Cells were treated with CDDP@PLs containing fluorescent dye FITC and free cisplatin, and after 4 h of incubation, cells were collected and washed three times with PBS (pH 7.4) and fixed with PBS containing 4% paraformaldehyde for 30 min at room temperature. Cells were washed again using PBS and then stained with DAPI (50 μg/mL) for 15 min. Cells were washed again and then mounted on Fisherbrand Superfrost^®^ microscope slides with Fluoromount G^®^ mounting buffer (Southern Biotech, Birmingham, AL, USA) for analysis via a confocal microscope (Olympus CX41) [28].

### 2.5. Stability Studies

Stability studies are very important in the development of liposomal formulations. For the approval and acceptance of pharmaceutical products, the continuation of the product’s safety, efficacy and quality are considered during their shelf life. The particle size, PDI and zeta potential with time are workable indicators of the stability of the liposomal suspension. The stability of the developed liposomes was carried out according to the international conference on harmonization (ICH) guidelines. The particle size analysis was measured, and 5 mL of each formulation was stored at 2–8 °C, room temperature (25 ± 2 °C), and elevated temperature (37 ± 2 °C) for three months. The particle size, PDI and zeta potential were then determined for the stability of the CDDP@PLs [28,29].

### 2.6. Acute Toxicity Study

Acute toxicity studies were performed according to the Organization for Economic Cooperation and Development (OECD) guidelines. The acute toxicity study was used to determine the safety and compatibility of the developed liposomal formulation components [30]. The main objective of the study was to determine the toxicity of the liposomal components and their distribution within the body. Twelve healthy albino mice were selected, acclimatized, efficiently monitored, divided into two groups, and kept in separate cages. The approval of the study was taken from the Pharmacy Animal Ethics Committee (PAEC), Institutional Ethical Committee under Reference No: 17-2020/PAEC. Distilled water was administered to the control group, and liposomal components were administered via a parenteral route to the test group at a single dose of 2000 mg/kg. Various parameters were properly monitored, such as physical observation, mortality rate, and food and water consumption. After two weeks, blood samples were collected from albino mice for blood biochemistry, and then the mice were sacrificed for histopathological examination of vital organs [31].

### 2.7. Statistical Analysis

GraphPad Prism 8 (GraphPad Software, San Diego, CA, USA), OriginPro 9.0, and Microsoft Excel were used for the statistical analysis of the data. All the experiments were carried out in triplicate and were expressed as the mean ± standard deviation (SD). One-way ANOVA followed by Post hoc Tukey analysis was applied to determine the statistical differences.

## 3. Results

### 3.1. Physicochemical Characterization of CDDP@PLs

#### 3.1.1. Particle Size, Polydispersity Index (PDI), and Zeta Potential

The particle size, PDI, and zeta potential of the CDDP@PLs are shown in Table 1. The particle size of all the formulations varied between 153.2 ± 3.08 nm and 206.4 ± 2.26 nm. The particle size decreased with the increase in the concentration of DOPE; this phenomenon could be due to the structural flexibility of DOPE. The particle size of the CDDP@PLs was ~200 nm, which plays an important role in the delivery of the drug to the target site. The size of the developed liposomes is suitable for parenteral administration and will deliver the drug to a tumor’s leaky vasculature via the enhanced permeation and retention (EPR) effect. The PDI of all the formulations were in the range of 0.261 to 0.422, which showed an acceptable size distribution, whereas the zeta potential ranged from −17.8 ± 1.26 mV to −24.6 ± 1.72 mV, showing acceptable stability of the liposomes.

#### 3.1.2. Entrapment Efficiency (E.E)

The entrapment efficiency of the CDDP@PLs formulations ranged from 47.25 ± 1.21 to 69.47 ± 1.23 (Table 1). The maximum E.E was observed for PL1 with minimum DOPE concentration, whereas the minimum E.E was observed for PL5 with maximum DOPE concentration. The entrapment efficiency decreased as the concentration of DOPE increased; this may be due to the structural flexibility of DOPE and the low transition temperature because the drug was not retained in the liposomes, leading to the decrease in the E.E of the liposomes. Similar effects were also reported by Jain, S. et al., 2021, [32], which also support the study with regard to the above-mentioned effect.

#### 3.1.3. Transmission Electron Microscopy (TEM)

**Figure 1:**
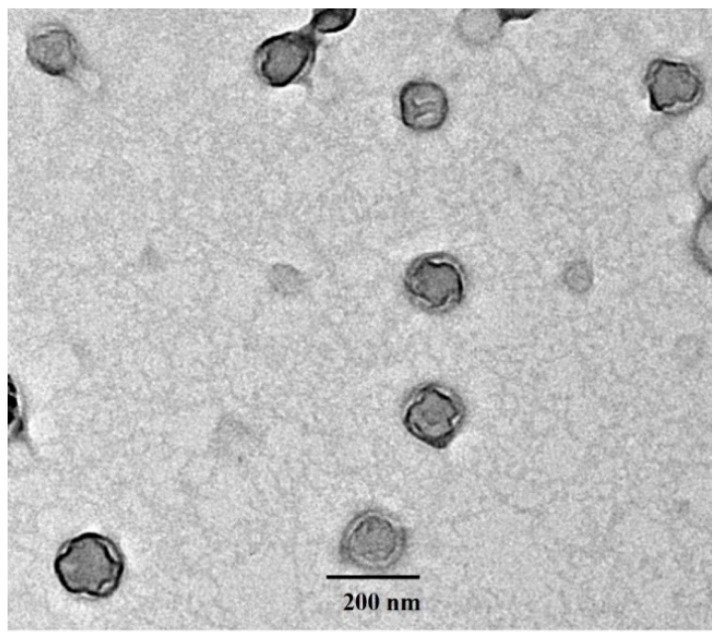
TEM image of the developed CDDP@PLs.

TEM images of the cisplatin-loaded pH-sensitive liposomes are shown in Figure 1. The size of the developed liposomes was ~200 nm, which is in accordance with the particle size of liposomes obtained via the DLS technique. Moreover, the TEM image revealed that vesicles were spherical with no sign of aggregation or fusion.

#### 3.1.4. Fourier Transform Infrared (FTIR) Spectroscopic Analysis

**Figure 2:**
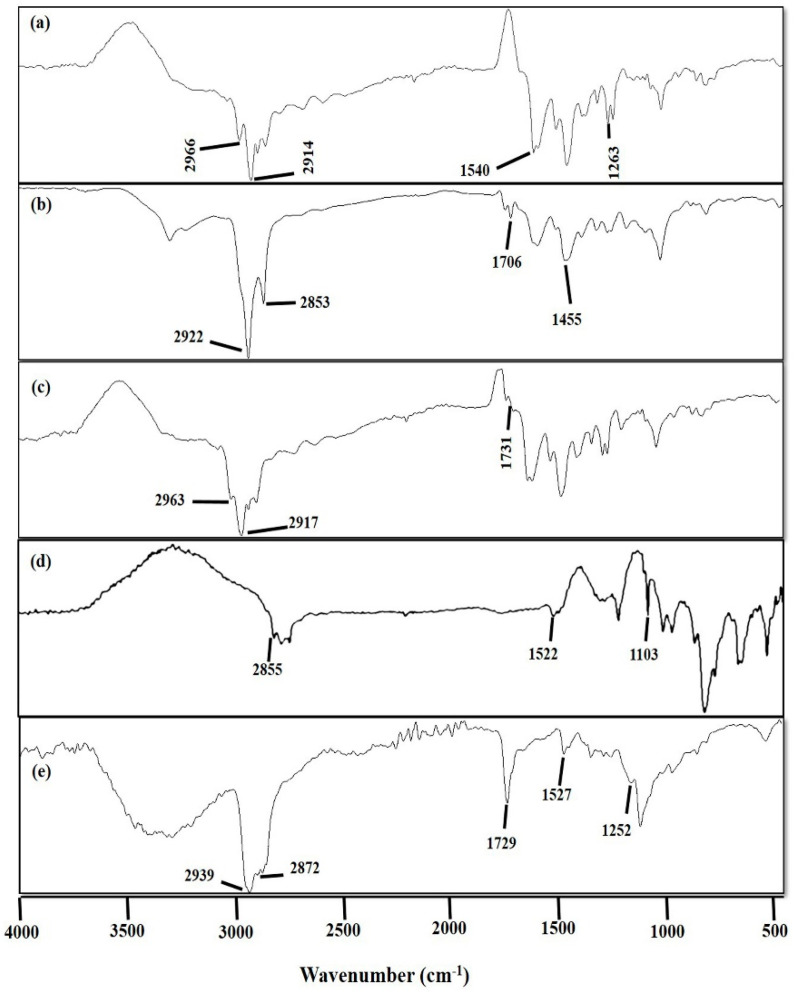
FTIR analysis of cisplatin (**a**), CHEMS (**b**), DOPE (**c**), DSPE-PEG_2000_ (**d**), and CDDP@PLs (**e**).

The FTIR spectra of cisplatin (Figure 2a) showed characteristic peaks at 1263 cm^−1^ (symmetric amine bending), 1540 cm^−1^ (asymmetric amine bending), 2914 cm^−1^ and at 2966 cm^−1^ (amine stretching) [33,34]. The FTIR spectra of CHEMS (Figure 2b) showed characteristic peaks at 1731 cm^−1^ (–C=O stretching vibration of ester and carboxyl group), 2917 cm^−1^ and 2963 cm^−1^ (–OH stretching vibration and out-of-plane bending vibration of a carboxyl group) [35]. The FTIR spectra of DOPE (Figure 2c) showed characteristic peaks at 1455 cm^−1^ (–CH bending), 1706 cm^−1^ (–C=O stretching), 2853 cm^−1^ (–OH stretching) and at 2922 cm^−1^ (–NH stretching). The FTIR spectra of DSPE-PEG_2000_ (Figure 2d) showed characteristic peaks at 2855 cm^−1^ (–CH stretching of alkane), 1522 cm^−1^ (N–O stretching) and at 1103 cm^−1^ (P=O stretching) [19,36], whereas the FTIR spectra of the CDDP@PLs (Figure 2e) showed characteristics peaks at 1252 cm^−1^ (1263 cm^−1^ of cisplatin due to symmetric amine bending), 1527 cm^−1^ (1540 cm^−1^ of cisplatin due to asymmetric amine bending), 1729 cm^−1^ (1731 cm^−1^ of CHEMS due to –C=O stretching vibration of ester and carboxyl group), 2872 cm^−1^ (2853 cm^−1^ of DOPE due to –OH stretching), and at 2939 cm^−1^ (2963 cm^−1^ due to –OH stretching vibration and out-of-plane bending vibration of the carboxyl group of CHEMS or due to 2966 cm^−1^ of cisplatin because of amine stretching). All the characteristic peaks of cisplatin were present in the liposomal formulation, indicating the compatibility of cisplatin and formulation components. Moreover, the FTIR analysis showed the physicochemical stability of CDDP@PLs [37].

#### 3.1.5. Differential Scanning Calorimetric (DSC) Analysis

**Figure 3:**
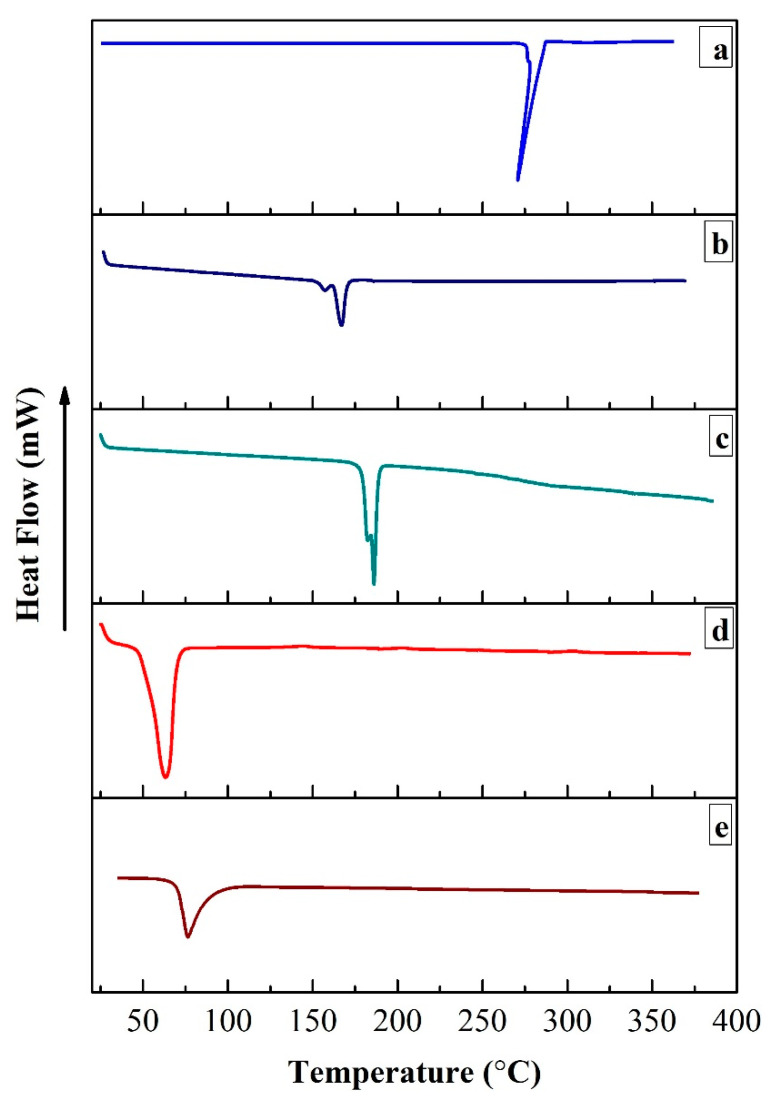
DSC analysis of cisplatin (**a**), CHEMS (**b**), DOPE (**c**), DSPE-PEG_2000_ (**d**), and CDDP@PLs (**e**).

Differential scanning calorimetric (DSC) analysis was performed to check any possible interaction and the crystalline/amorphous nature of the cisplatin, CHEMS, DOPE, DSPE-PEG_2000_ and CDDP@PLs, as shown in Figure 3. The melting-point-based endothermic peak of cisplatin was shown at 270 °C, CHEMS at 168 °C, DOPE at 186 °C, and DSPE-PEG_2000_ at 64 °C. The CDDP@PLs did not show any cisplatin-based endothermic peak in the range of 250 to 300 °C, indicating that the drug was present in the amorphous or molecular dispersion in the vesicles. The amorphous nature of the drug in the CDDP@PLs indicates the enhancement in the dissolution profile, which ultimately provides enhanced availability at the site of action [38,39].

#### 3.1.6. In Vitro Release and Kinetic Modeling

**Figure 4:**
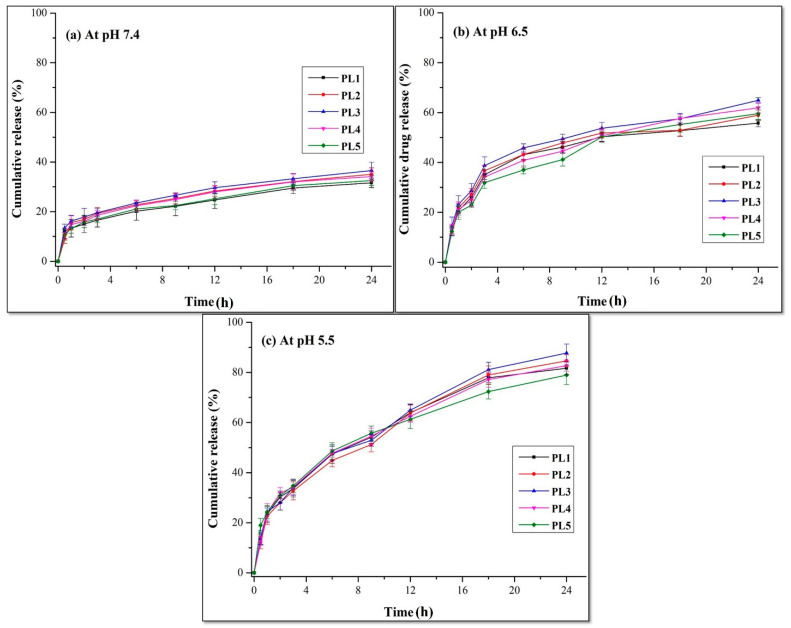
In vitro release profile of CDDP@PLs at pH 7.4 (**a**), 6.5 (**b**) and at 5.5 (**c**).

The in vitro release of the CDDP@PLs was performed in PBS (pH 7.4, 6.5 and 5.5) (Figure 4). At pH 7.4, the release rate of cisplatin from liposomes was less than 40%, whereas the rate was almost 65% at a pH of 6.5, and at a pH of 5.5, more than 80% cisplatin was released from the liposomes within 24 h. The cisplatin release rate at pH 5.5 indicates the rapid destabilization of the liposomes in the acidic tumor microenvironment. Moreover, at the physiological pH (bloodstream), the pH-responsive lipids were not protonated and remained intact, but after endocytosis, the pH in the endosome dropped, and protonation of the lipids eroded the liposomes, leading to the release of the drug [40,41].

The in vitro release can be affected by the concentration of CHEMS (cholesterol derivative). By increasing the concentration of CHEMS (in PL1, PL2 and PL3), the amount of cisplatin released from CDDP@PLs was decreased. This decrease in the release rate was observed with a higher concentration of CHEMS and optimum DOPE concentration, which might be due to slower diffusion of cisplatin from the lipid bilayer. The maximum release of 87% was observed by PL3 with optimum DOPE concentration, and above this level (in PL4 and PL5), the release was further decreased with the increased concentration of DOPE [42]. The values of kinetic modeling (at pH 7.4, 6.5 and 5.5) showed that the cisplatin release from the liposomes was best fit to a Korsmeyer–Peppas model, which is usually followed by liposomes [43]. The value of release exponent for the liposomes at pH 7.4 and 6.5 were less than 0.45, indicating the mechanism of drug release by Fickian diffusion, whereas in the case of a pH of 5.5, all the formulations showed the same mechanism, except for in the case of PL2 and PL3, which were greater than 0.45, indicating the mechanism of non-Fickian diffusion or anomalous behavior drug release (Table 2).

### 3.2. Cytotoxicity Study

**Figure 5:**
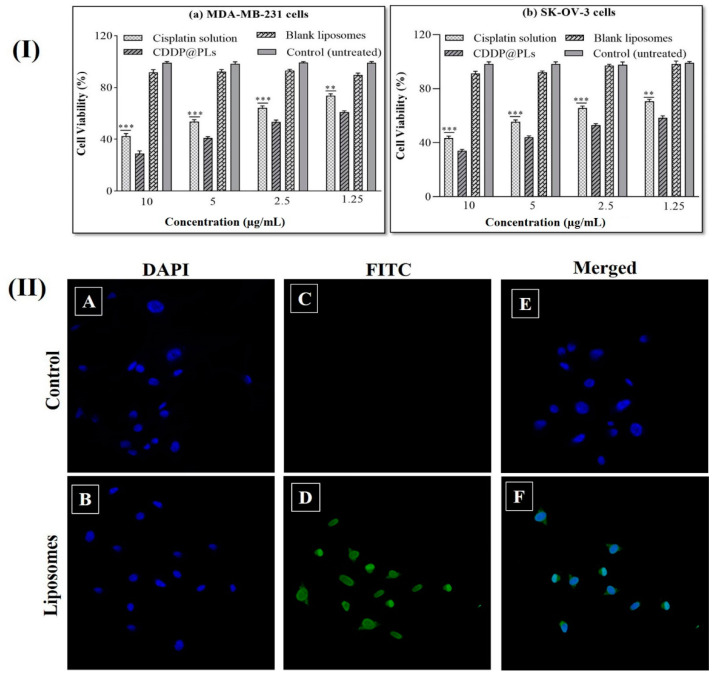
(**I**) Cytotoxicity study of the cisplatin solution and CDDP@PLs on (**a**) MDA-MB-231 cells and (**b**) SK-Ov-3 cells. Data are presented as mean ± SD (*n* = 3). ** *p* < 0.01, *** *p* < 0.001 and (**II**) cell uptake of FITC by the MDA-MB-231 cell line.

The cytotoxicity potential of CDDP@PLs was evaluated via an MTS assay in comparison with cisplatin solution against previously cultured MDA-MB-231 and SK-OV-3 cells. The cells in 96-well plates were applied with cisplatin solution and CDDP@PLs and then incubated for 24 h at 37 °C. It was depicted from the cytotoxicity profile that CDDP@PLs showed greater cytotoxicity as compared to cisplatin solution, indicating the improved killing of the cells as compared to the cisplatin solution. The blank liposomes had no effect on the cell cytotoxicity, indicating the biocompatibility of the liposomes. The CDDP@PLs showed higher toxicity towards both cancer cell lines and decreased viability after 24 h (Figure 5I) [44,45]. 

### 3.3. Cell Uptake Studies

A qualitative cellular uptake study was performed via confocal scanning laser microscopy (CSLM) to observe the colocalization of FITC-labeled CDDP@PLs, as shown in Figure 5II). The cells were treated with DAPI; a blue color was observed in cells due to nucleus staining with DAPI. Strong bright fluorescence was observed in cells treated with FITC-labeled liposomes, while no fluorescence was observed in control. The presence of green fluorescence confirmed the uptake of liposomes by the cells. CSLM was also used to observe the binding and internalization of liposomes in MDA-MB-231 cell lines. DAPI produces a blue color when interacting in cells, whereas FITC produces a green color after interaction with the DNA of the cell. The improved internalization within the cells and nuclei might be due to the presence of biocompatible lipids present in the liposomes. The study performed by Chen, Y. et al., 2013, [46] also supports findings of the cellular uptake study by the qualitative technique for DOPE pH-sensitive liposomes.

### 3.4. Stability Study

The stability of the CDDP@PLs was determined as shown in Table 3. The liposomal formulations were stored at different temperatures, i.e., 2–8 °C, room temperature (25 ± 2 °C), and at elevated temperature (37 ± 2 °C) for three months. After three months of storage, the developed CDDP@PLs showed a slight increase in the particle size and PDI and a decrease in the zeta potential. The particle size, PDI, and zeta potential showed no significant difference at 2–8 °C and room temperature (25 ± 2 °C) (*p* < 0.05). However, there was slight increase in the size, PDI and zeta potential at elevated temperature (37 ± 2 °C), which was due to the effect of elevated temperature on the phosphatidylethanolamine contents in the liposomes. The stability study could demonstrate the good stability of the developed CDDP@PLs [22,29].

### 3.5. Acute Toxicity Study

**Figure 6:**
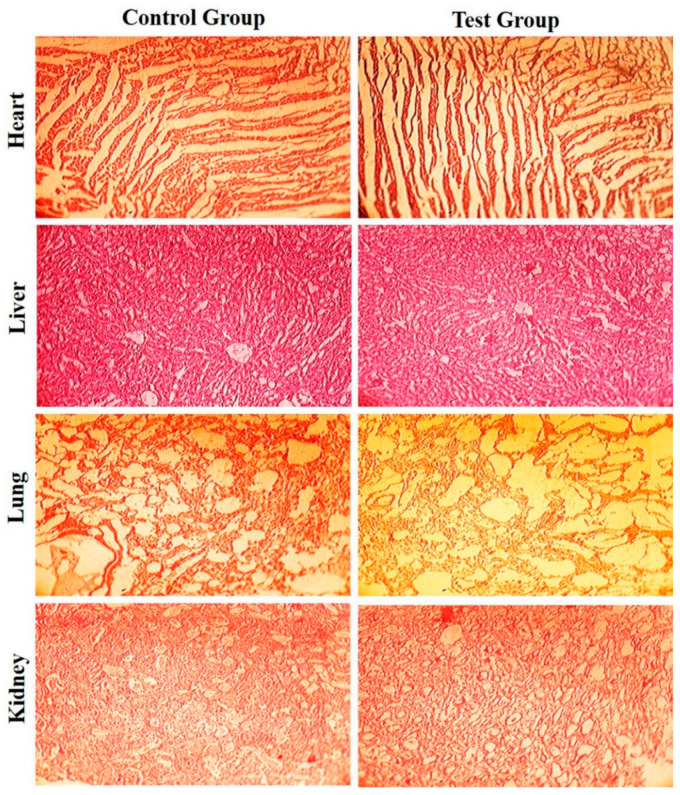
Histopathological examination for different organs of control and test group.

An acute toxicity study was performed to evaluate the safety of the carriers/liposomal components and biocompatibility in the biological system. Albino mice were slaughtered, and vital organs were removed and dipped in 10% formalin solution. Various biochemical and hematological parameters of the albino mice were monitored, as illustrated in Table 4. The various parameters of the test group showed slight variation as compared to the control group. No mortality rate was observed during the study, there was no significant nor any gross histopathological lesions in the vital organs, as shown in Figure 6. Moreover, there were no signs of lesions, disruption or deformation and any type of pathological changes within the vital organs of the test group in comparison with the control group. The lack of changes shows the safety and biocompatibility of the liposomal excipients [32,47,48].

## 4. Conclusions

In the present study, CDDP@PLs were fabricated and evaluated for physicochemical characterization, in vitro release profile, in vitro cytotoxicity, cell uptake study, stability study and acute toxicity study. Our results advocate that cisplatin, when encapsulated into pH-responsive liposomes, is effective in delivering drugs at tumor sites. Liposomes with particle sizes lower than 200 nm facilitate higher drug concentrations in the tumor microenvironment and poor lymphatic drainage by increasing the therapeutic effect via the enhanced permeation and retention (EPR) effect. Mechanistically, the pH-responsive liposomes undergo rapid destabilization in an acidic environment and release the drug at the tumor site. The neutral cone-shaped DOPE and weakly acidic amphiphile (CHEMS) were used for the fabrication of CDDP@PLs due to their fusogenic behavior in the lipid bilayer. Further, the developed CDDP@PLs will deliver the drug to the tumor neo-vascularization due to small-sized liposomes, tumor-specific improved cytotoxicity, and enhanced cellular internalization. The acute toxicity study performed in albino mice showed the safety and biocompatibility of the developed pH-responsive carrier system. Finally, the developed CDDP@PLs provide better tumor microenvironment responsive release faster at a pH of 5.5 than at a pH of 7.4 and 6.5, which provides a better perspective regarding a safe and effective tumor-targeting lipid-vesicles-based drug delivery system that will maximize the therapeutic effect and minimize the dose-related toxicity of cisplatin.

## Figures and Tables

**Table 1:** Physicochemical characteristics of CDDP@PLs.

Code	Lipid Mixture Ratio (DOPE: CHEMS: DSPE:PEG_2000_)	Lipid Mixture Weight (mg) DOPE: CHEMS: DSPE-PEG_2000_	Cisplatin (mg)	Particle Size (nm)	PDI	Zeta Potential (mV)	(%) E.E
PL1	45:50:05	33.48:24.33:14.02	5.0	206.4 ± 2.26	0.417 ± 0.008	−24.6 ± 1.72	69.47 ± 1.23
PL2	50:45:05	37.20:21.90:14.02	5.0	194.3 ± 2.21	0.422 ± 0.010	−22.8 ± 2.01	65.52 ± 2.14
PL3	55:40:05	40.92:19.46:14.02	5.0	191.2 ± 1.67	0.386 ± 0.009	−22.5 ± 0.38	61.23 ± 1.98
PL4	60:35:05	44.64:17.03:14.02	5.0	171.9 ± 2.26	0.371 ± 0.011	−20.2 ± 2.69	52.19 ± 1.45
PL5	65:30:05	48.36:14.60:14.02	5.0	153.2 ± 3.08	0.261 ± 0.007	−17.8 ± 1.26	47.25 ± 1.21

**Table 2:** Kinetic modeling of in vitro release profile of CDDP@PLs at pH 7.4, 6.5 and 5.5.

Formulation Code	pH	Zero Order	First Order	Higuchi Model	Korsmeyer–Peppas Model	
		R^2^	R^2^	R^2^	R^2^	N
PL1	7.4	0.0072	0.1690	0.8103	0.9898	0.279
	6.5	0.0837	0.4866	0.8409	0.9690	0.309
	5.5	0.5912	0.9015	0.9823	0.9949	0.426
PL2	7.4	0.0728	0.2674	0.8440	0.9910	0.279
	6.5	0.0778	0.4938	0.8405	0.9731	0.307
	5.5	0.6863	0.9278	0.9915	0.9940	0.464
PL3	7.4	0.0734	0.1488	0.7930	0.9952	0.271
	6.5	0.1466	0.5724	0.8670	0.9823	0.316
	5.5	0.6707	0.9242	0.9904	0.9943	0.456
PL4	7.4	0.0297	0.2282	0.8335	0.9968	0.289
	6.5	0.3215	0.6595	0.9233	0.9910	0.349
	5.5	0.5821	0.8919	0.9803	0.9946	0.421
PL5	7.4	0.0759	0.2496	0.8439	0.9936	0.294
	6.5	0.3830	0.6810	0.9375	0.9900	0.364
	5.5	0.4436	0.8238	0.9582	0.9988	0.377

**Table 3:** Stability study of CDDP@PLs at different temperature (after 3 months).

Formulation	Storage Condition	Time	Particle Size (nm)	PDI	Zeta Potential (mV)
PL1	Initial		206.4 ± 2.26	0.417 ± 0.008	−24.6 ± 1.72
	2–8 °C	After 90 days	209.5 ± 1.78	0.423 ± 0.004	−23.2 ± 1.07
	25 °C		214.7 ± 2.45	0.441 ± 0.005	−22.8 ± 0.86
	37 °C		219.8 ± 1.96	0.446 ± 0.003	−22.3 ± 1.01
PL2	Initial		194.3 ± 2.21	0.422 ± 0.010	−22.8 ± 2.01
	2–8 °C	After 90 days	197.1 ± 1.41	0.429 ± 0.011	−22.3 ± 0.61
	25 °C		199.2 ± 0.97	0.434 ± 0.008	−21.7 ± 0.70
	37 °C		204.7 ± 0.92	0.440 ± 0.005	−20.9 ± 1.40
PL3	Initial		191.2 ± 1.67	0.386 ± 0.009	−22.5 ± 0.38
	2–8 °C	After 90 days	196.3 ± 0.52	0.388 ± 0.004	−22.2 ± 1.87
	25 °C		198.8 ± 1.41	0.391 ± 0.006	−21.7 ± 0.56
	37 °C		201.4 ± 1.35	0.398 ± 0.013	−21.3 ± 1.02
PL4	Initial		171.9 ± 2.26	0.371 ± 0.011	−20.2 ± 2.69
	2–8 °C	After 90 days	173.6 ± 2.08	0.372 ± 0.007	−19.5 ± 1.63
	25 °C		174.7 ± 1.15	0.376 ± 0.009	−19.3 ± 2.07
	37 °C		182.9 ± 1.14	0.81 ± 0.013	−18.8 ± 0.91
PL5	Initial		153.2 ± 3.08	0.261 ± 0.007	−17.8 ± 1.26
	2–8 °C	After 90 days	156.7 ± 1.85	0.264 ± 0.003	−17.3 ± 1.21
	25 °C		159.2 ± 2.92	0.267 ± 0.006	−16.5 ± 1.56
	37 °C		163.4 ± 1.60	0.273 ± 0.010	−16.2 ± 2.17

**Table 4:** Biochemical and hematological parameters of albino mice.

**Biochemical Parameters**	**Control Group**	**Test Group**
Bilirubin (mg/dL)	0.58 ± 0.09	0.61 ± 0.13
Urea (mg/dL)	35.45 ± 1.98	34.21 ± 2.34
Creatinine (mg/dL)	0.19 ± 0.12	0.23 ± 0.16
Uric acid (mg/dL)	2.07 ± 0.45	2.10 ± 0.39
Cholesterol (mg/dL)	59.87 ± 3.44	61.22 ± 2.87
Triglycerides (mg/dL)	74.32 ± 1.34	72.10 ± 1.65
ALT(IU/L)	69.55 ± 1.23	70.98 ± 0.97
Alkaline Phosphatase (IU/L)	486.43 ± 4.59	493.29 ± 4.11
**Hematological Parameters**	**Control Group**	**Test Group**
Red blood cells	5.48 ± 0.67	5.21 ± 0.84
White blood cells	7.67 ± 0.98	7.58 ± 0.77
Platelets	4.24 ± 0.59	4.21 ± 0.68
Lymphocytes	61.42 ± 1.33	60.34 ± 1.19
Monocytes	1.53 ± 1.90	1.49 ± 1.76
Neutrophils	44.32 ± 0.88	45.90 ± 0.93
Mean corpuscular volume (MCV)	63.56 ± 0.61	62.87 ± 0.74
Mean corpuscular hemoglobin	21.33 ± 0.56	22.04 ± 0.43
Hemoglobin (g/dL)	12.1 ± 0.35	12.5 ± 0.29

## Data Availability

The data presented in this study are openly available.
